# Affective psychosis, Hashimoto's thyroiditis, and brain perfusion abnormalities: case report

**DOI:** 10.1186/1745-0179-3-31

**Published:** 2007-12-20

**Authors:** Alberto Bocchetta, Giorgio Tamburini, Pina Cavolina, Alessandra Serra, Andrea Loviselli, Mario Piga

**Affiliations:** 1 Sezione di Farmacologia Clinica, Dipartimento di Neuroscienze Bernard B Brodie, Università di Cagliari, Azienda Ospedaliero-Universitaria di Cagliari, via Ospedale 46, 09124 Cagliari, Italy; 2 Sezione di Neurologia, Dipartimento di Scienze Cardiovascolari e Neurologiche, Università di Cagliari, Azienda Ospedaliero-Universitaria di Cagliari, via Ospedale 46, 09124 Cagliari, Italy; 3 Sezione di Neuropsichiatria Infantile Dipartimento di Neuroscienze Bernard B Brodie, Università di Cagliari, Azienda Ospedaliero-Universitaria di Cagliari, via Ospedale 119, 09124 Cagliari, Italy; 4 Medicina Nucleare, Dipartimento di Scienze Mediche Internistiche Mario Aresu, Università di Cagliari, Azienda Ospedaliero-Universitaria di Cagliari, S.P. Monserrato-Sestu, 09042 Monserrato, Cagliari, Italy; 5 Endocrinologia, Dipartimento di Scienze Mediche Internistiche Mario Aresu, Università di Cagliari, Azienda Ospedaliero-Universitaria di Cagliari, S.P. Monserrato-Sestu, 09042 Monserrato, Cagliari, Italy

## Abstract

**Background:**

It has recently become evident that circulating thyroid antibodies are found in excess among patients suffering from mood disorders. Moreover, a manic episode associated with Hashimoto's thyroiditis has recently been reported as the first case of bipolar disorder due to Hashimoto's encephalopathy. We report a case in which Hashimoto's thyroiditis was suspected to be involved in the deteriorating course of mood disorder and discuss potential pathogenic mechanisms linking thyroid autoimmunity with psychopathology.

**Case presentation:**

A 43-year-old woman, with a history of recurrent depression since the age of 31, developed manic, psychotic, and soft neurological symptoms across the last three years in concomitance with her first diagnosis of Hashimoto's thyroiditis. The patient underwent a thorough medical and neurological workup. Circulating thyroperoxidase antibodies were highly elevated but thyroid function was adequately maintained with L-thyroxine substitution. EEG was normal and no other signs of current CNS inflammation were evidenced. However, brain magnetic resonance imaging evidenced several non-active lesions in the white matter from both hemispheres, suggestive of a non-specific past vasculitis. Brain single-photon emission computed tomography showed cortical perfusion asymmetry particularly between frontal lobes.

**Conclusion:**

We hypothesize that abnormalities in cortical perfusion might represent a pathogenic link between thyroid autoimmunity and mood disorders, and that the rare cases of severe Hashimoto's encephalopathy presenting with mood disorder might be only the tip of an iceberg.

## Background

A recent twin study has supported the hypothesis that autoimmune Hashimoto's thyroiditis may be part of the genetic vulnerability (or an endophenotype) for bipolar disorder [1]. The twin study was prompted by the previous report of circulating thyroperoxidase antibodies (TPO-Abs) in 28% of 226 bipolar outpatients participating in the Stanley Foundation Bipolar Network in the United States and the Netherlands compared with 13% of controls [2].

Here we describe a case of a patient with bipolar psychosis and Hashimoto's thyroiditis who underwent a thorough medical and neurological workup. We found abnormalities in cortical perfusion that we hypothesize might represent a pathogenic link between thyroid autoimmunity and mood disorders.

## Case presentation

The patient, a 43-year-old housewife, first came to the outpatient unit of the department of neurosciences in 2005. She had suffered from mood disorder since the age of 31 and had been treated repeatedly with antidepressants across the following 9 years. Her level of functioning prior to illness had been adequate, subsequently returning to a similar level after recovery from episodes. During a hospitalization at a dermatology unit at age 37 for urticaria vasculitis, an endocrinological consult led to the first diagnosis of Hashimoto's thyroiditis. On palpation, thyroid had been found increased in volume and hard-elastic in consistency. Ultrasound had revealed increased volume of the gland and a diffuse non-homogeneous echopattern. TPO-abs were abnormally elevated, while thyroglobuline antibodies (TG-Abs) were normal. Thyroid function tests had evidenced subclinical hypothyroidism (TSH = 3.68 IU/ml; normal FT3, and low FT4 = 0.74 ng/dl, with normal range 1.0–1.8 ng/dl). Then, substitution therapy with L-thyroxine was started for subclinical hypothyroidism. The course of mood disorder, after the first 9 years of recurrent episodes of major depression, had worsened over the last 3 years, when manic and psychotic symptoms became manifest, even in the absence of ongoing antidepressant treatment. In particular, 4 hospitalizations had been necessary due to severe episodes of both polarities. According to the hospital records provided, the latter episodes were characterized, among other symptoms, by marked anxiety, agitation, somatic complaints, transient persecutory delusions, and suicidal thoughts. Hospital diagnoses varied from bipolar disorder I, mixed to bipolar disorder I, depressive, to affective psychosis, and, last, to schizoaffective disorder. Since her first diagnosis of bipolar disorder at age 41, Ms. A had been treated with various regimens including valproate, benzodiazepines, typical (haloperidol) and atypical (quetiapine, risperidone, amisulpiride) antipsychotics. Carbamazepine had been stopped after a brief trial because of side effects (nausea and vomiting), whereas lithium had been until then considered contraindicated by hypothyroidism.

When first seen at the department of neurosciences, the patient's symptoms included depressed mood, psychomotor retardation, suicidal thoughts, and persecutory ideas. Moreover, she manifested somnolence, fatigue, nausea, and ataxia, attributable in part to side effects from her current mood-stabilizing treatment (sodium valproate, 900 mg daily). The patient was also taking daily chlordemethyldiazepam (3 mg), L-thyroxine (0.075 mg), whereas risperidone, prescribed during last hospitalization, had been stopped 4 weeks earlier. Serum valproate concentration was found within the therapeutic range (50.4 μg/ml). Thyroid hormone replacement with L-thyroxine was adequate, as serum concentrations of free triiodothyronine and thyroxine were normal, as was thyroid stimulating hormone (TSH) (1.24 IU/ml). TPO-Abs were highly elevated (>1000 mU/ml; normal range < 35 mU/ml), while TG-Abs were normal.

Mood-stabilizing medication was changed. Lithium carbonate was added and increased gradually, while sodium valproate was tapered and stopped over a few weeks. Ataxia, somnolence, nausea, and psychomotor retardation ameliorated, but the patient manifested anxiety and agitation in addition to the pre-existing depressed mood and persecutory ideas. Chlordemethyldiazepam was substituted with lorazepam and low-dose haloperidol was prescribed.

During a subsequent visit at the department for measurement of lithium serum concentration, the patient, seemingly only slightly tense at entry, suddenly started to laugh inappropriately and to cry, maintaining that someone was coming to kill her. Haloperidol was administered (1 mg orally) and symptoms promptly resolved. She admitted that her behavior had been prompted by visual hallucinations ("I have seen dark strangers at the door coming to take me away"). Similar crises occurred at home over the following weeks and were treated with low doses of promazine.

A few weeks later, the patient manifested an episode of fainting, accompanied with involuntary limb movements. She was taken to an emergency room where neurological assessment including EEG ruled out epileptic seizures and other prominent diseases. Subsequently, the patient reported weakness, gait problems, and transient involuntary movements of the hands (described as myoclonus). A neurological examination evidenced only moderate reduction of synkinetic movements of the arms during walking and mild hypermetry of the finger-nose test on the right side, interpreted by the neurology consultant as drug-related side-effects. Current psychotropic daily treatment was lithium carbonate 900 mg, lorazepam 7.5 mg, promazine (72 mg). Lithium serum concentration (12 hours after last administration) was 0.57 mmol/L.

Lithium treatment was maintained, also given high suicide risk, both personal (recurrent suicidal thoughts) and familial (two first-degree relatives had committed suicide).

The nature of neurological symptoms, prominent despite relatively low doses of medication, remained to be clarified. Fainting, gait problems, and myoclonus might have been "functional" in nature, as those seen in conversion or somatization disorder; nevertheless, diagnostic procedures aiming at ruling out organic causes were in order. In particular, as Hashimoto's thyroiditis is often associated with other autoimmune disorders, a comprehensive medical workup was initiated. Thyroid echographic scan evidenced reduced thyroid size (estimated total volume of 5.1 cubic centimeters), with a diffusely non-homogeneous echo-pattern, consistent with previous diagnosis of Hashimoto's thyroiditis. No abnormalities were found in markers of current inflammation and vasculitis, and in the following antibodies: anti-nuclear, anti-extractable nuclear antigens (anti-ribonucleic protein, anti-Smith, anti-SSA, anti SSB, anti Jo1, anti-Scl-70), anti-DNA, anti-neutrophil cytoplasmic (c-ANCA and p-ANCA), anti-cardiolipin, and anti-glutamic acid decarboxylase A. Nailfold capillaroscopy evidenced a reduced number of capillaries and an irregularity in pattern, consistent with past vasculitis.

From a neurological point of view, multiple sclerosis was the first organic cause to be investigated, being highly prevalent in Sardinia and often associated with other autoimmune disorders, including Hashimoto's thyroiditis. Visual, acoustic, and somato-sensorial evoked potentials were found normal.

Brain magnetic resonance imaging (MRI) evidenced several small areas of high signal intensity in the white matter from both hemispheres, in particular in sub-cortical frontal sites. Such areas were not enhanced with paramagnetic contrast; i.e. were unlikely to be active lesions. They were suggestive of a non-specific past vasculitis.

The neurology consultant, given the evoked potentials and MRI findings, did not recommend further diagnostic procedures for multiple sclerosis, such as a lumbar puncture in the search of immunoglobulin oligoclonal bands.

**Figure 1:**
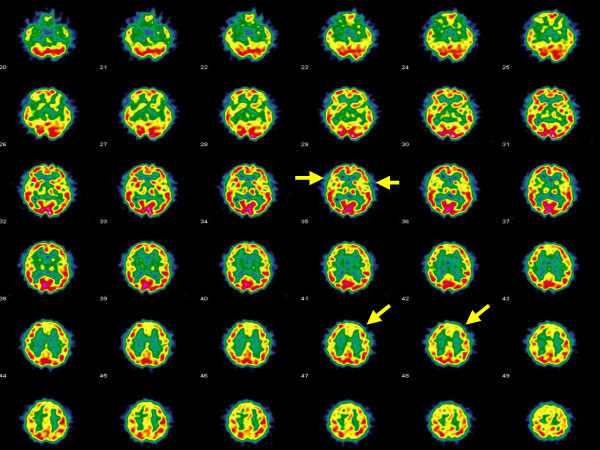
Brain SPECT transaxial images showing diffuse patchy cortical distribution of 99 mTc-ECD, in particular significant cortical hypoperfusion in the left frontal lobe and in both temporal lobes (arrows).

Brain single-photon emission computed tomography (SPECT) was requested to better explore cortical perfusion and function. Thirty minutes after intravenous injection of the radiopharmaceutical (99 mTc-ethyl cysteinate dimer – ECD) the patient underwent brain SPECT. The imaging analysis showed relevant irregularity in cortical distribution of the tracer regarding both hemispheres (Figure 1). Cortical perfusion asymmetry was particularly evidenced between frontal lobes (reduced uptake by the left side). Areas of reduced cortical uptake were also evidenced in both superior gyri of temporal lobes. ECD distribution was also irregular in basal ganglia while resulted normal in both cerebellar hemispheres.

The conclusion from overall medical workup was that the patient might have suffered from vasculitis, with traces left in periphery (as seen by capillaroscopy) and in cerebral areas (as seen by MRI). Moreover, she was currently experiencing abnormalities in cortical function, particularly in frontal lobes, as evidenced by SPECT imaging.

Over the 12 months following the first visit at the department, the patient gradually stabilized with maintenance with lithium, olanzapine, and benzodiazepines. Functioning improved although not returning to pre-morbid levels. Neuropsychological testing (WAIS-R) placed the patient in the borderline range of intellectual functioning, mostly due to impairment in memory retrieval.

## Discussion

Abnormalities in thyroid function have been long associated with psychiatric symptoms, but it has recently become evident that circulating thyroid antibodies, even in the absence of hormone abnormalities, are found in excess among patients suffering from mood and anxiety disorders [2,3]. A manic episode associated with Hashimoto's thyroiditis, pathological EEG and response to short-term treatment with high doses of prednisolone, has recently been reported as the first case of bipolar disorder due to Hashimoto's encephalopathy [4]. The latter is a rare severe syndrome with different clinical presentations and course (for review see [5]). Onset may be acute or subacute. Presentation may include alteration of conscious level, seizures, tremor, myoclonus, ataxia, or multiple stroke-like episodes. Psychiatric symptoms, including depression and psychosis, have also been reported [6,7]. Course of Hashimoto's encephalopathy may be relapsing/remitting or progressive, even evolving into dementia. Nonspecific imaging abnormalities may be present. Brain MRI findings may change abruptly and drastically. For example, reversible MRI lesions in the cerebral white matter, supposedly reflecting brain edema, have been reported in one case where antithyroid antibodies were detected in the cerebrospinal fluid [8]. Even if the pathophysiology is unknown, the focal and global cerebral involvement have been attributed to autoimmune-mediated cerebral vasculitis, with or without immune complex deposition [9,10], and an anti-neuronal antibody-mediated mechanism [11].

With regard to brain SPECT imaging, either diffuse hypoperfusion not related to specific region [12,13] or focal hypoperfusion [14] have been described in Hashimoto's encephalopathy. Similar brain SPECT patterns have been found in other immunomediated diseases presenting with neurological and psychiatric symptoms, such as systemic lupus erythematosus, as the probable expression of autoimmune involvement of brain due to vasculitis and/or anti-neuron autoantibodies [15].

A limitation of this case report is that we cannot prove causality between Hashimoto's thyroiditis and abnormalities in cortical perfusion and cannot rule out that the described association was only by chance, considering the high prevalence of Hashimoto's thyroiditis. However, it is noteworthy that cortical hypoperfusion was also found in brain SPECT imaging from series of patients with euthyroid autoimmune thyroiditis, even in the absence of any treatment or clinical evidence of CNS involvement [16,17]. Abnormalities were reported either with no topographic pattern [16] or with preferential localization in the frontal lobes [17]. In their series of patients with euthyroid thyroiditis, Zettinig and coworkers [16] found no association with depression and concluded that the presence of cerebral vasculitis was the most likely pathogenic model explaining cerebral hypoperfusion, as already found in cases of Hashimoto's encephalopathy.

## Conclusion

We hypothesize that abnormalities in cortical perfusion might represent a pathogenic link between Hashimoto's thyroiditis and mood disorders, even in the absence of other prominent symptoms of current CNS inflammation or EEG abnormalities, as in the described case. Accordingly, the rare severe cases of Hashimoto's encephalopathy presenting with mood disorder [4,6,7] may represent only the tip of an iceberg.

## Competing interests

The author(s) declare that they have no competing interests.

## Authors' contributions

AB first observed the case, coordinated the clinical workup, took care of patient's treatment and follow up, conceived the case report, and drafted the manuscript, GT carried out and interpreted the neurological workup, PC performed and interpreted the neuropsychological testing, AS performed and discussed the brain SPECT imaging, AL coordinated the medical and endocrinological workup and helped to draft the manuscript, MP coordinated the performance and interpretation of brain SPECT imaging and helped to draft the manuscript. All authors read and approved the final manuscript.
